# Integration of a Thermoelectric Heating Unit with Ionic Wind-Induced Droplet Centrifugation Chip to Develop Miniaturized Concentration Device for Rapid Determination of Salmonella on Food Samples Using Antibody-Functionalized SERS Tags

**DOI:** 10.3390/s20247177

**Published:** 2020-12-15

**Authors:** Yi-Jhen Chen, Yuan-Yu Chen, Kai-Hao Wang, Chih-Hsien Wang, Chiou-Ying Yang, Lai-Kwan Chau, Shau-Chun Wang

**Affiliations:** 1 Department of Chemistry and Biochemistry, National Chung Cheng University, Chia-Yi 621, Taiwan; yijhen110183@gmail.com (Y.-J.C.); curtis992250@gmail.com (Y.-Y.C.); howard817102@gmail.com (K.-H.W.); 2 Center for Nano Bio-Detection, Advanced Institute of Manufacturing with High-tech Innovations (AIM-HI), National Chung Cheng University, Chia-Yi 621, Taiwan; jshian7141@gmail.com; 3 Institute of Molecular Biology, National Chung Hsing University, Taichung 402, Taiwan; cyyang@dragon.nchu.edu.tw

**Keywords:** microfluidics, ionic wind, thermoelectric drying, microcentrifuge, SERS tag, bacteria detection, food sample

## Abstract

When a centrifugation-enriched sample of 100 μL containing the surface-enhanced Raman scattering (SERS) tag-bound bacteria (Salmonella in this study) is siphoned onto a glass slide next to an embedded thermoelectric heating chip, such a sessile droplet is quickly evaporated. As the size of the sample droplet is significantly reduced during the heating process, ionic wind streams from a corona discharge needle, stationed above the sample, sweep across the liquid surface to produce centrifugal vortex flow. Tag-bound Salmonella in the sample are then dragged and trapped at the center of droplet bottom. Finally, when the sample is dried, unlike the “coffee ring” effect, the SERS tag-bound Salmonella is concentrated in one small spot to allow sensitive detection of a Raman signal. Compared with our previous electrohydrodynamic concentration device containing only a corona discharge needle, this thermoelectric evaporation-assisted device is more time-effective, with the time of concentrating and drying about 100 μL sample reduced from 2 h to 30 min. Hence, sample throughput can be accelerated with this device for practical use. It is also more sensitive, with SERS detection of a few cells of Salmonella in neat samples achievable. We also evaluated the feasibility of using this device to detect Salmonella in food samples without performing the culturing procedures. Having spiked a few Salmonella cells into ice cubes and lettuce leaves, we use filtration and ultracentrifugation steps to obtain enriched tag-bound Salmonella samples of 200 μL. After loading an aliquot of 100 μL of sample onto this concentration device, the SERS tag signals from samples of 100 g ice cubes containing two Salmonella cells and 20 g lettuce leaf containing 5 Salmonella cells can be successfully detected.

## 1. Introduction

Conventional pathogenic bacteria tests require tedious cultivation procedures because microorganism counts are below 10^3^ colony forming units (CFU) per mL in typical biological samples from living sources. In particular, the food safety standard imposed by the administrative authority demands an even stricter level. For instance, Salmonella tolerance in ready-to-eat food products is less than a single pathogen [1]. The culturing time to determine Salmonella often exceeds one day. Although alternative pathogen determination methods using nucleic acid probes to perform polymerase chain reaction (PCR) amplification procedures can reduce testing times to hours, PCR-based tests, going through repetitive heating-and-cooling cycles, have to be carried out by well-trained technicians with sophisticated equipment in a centralized laboratory.

Recently isothermal nucleic acid amplification tests have emerged as a popular means to determine food pathogen bacteria such as Salmonella. The reported results of detecting Salmonella in food samples use a loop-mediated isothermal amplification method, recombinase polymerase amplification method, and thermophilic helicase-dependent method [2,3,4,5,6]. Because these methods can be carried out at constant temperature, heating-and-cooling steps are not required. Testing devices are much simpler and usually portable, suitable to point-of-care test (POCT) purposes. However, the specificity of isothermal amplification methods is not ideal in general. These semi-quantitative methods sometimes result in unsatisfactory detection.

Electrochemical immunosensors or aptasensors have been employed to detect Salmonella in the past decade [7,8]. With sample concentration steps using antibody-functionalized magnetic nanoparticles, a recent study showed that the detection limit was 50 CFU mL^−1^ [9]. When the detection signal was amplified through enzymatic reactions, the detection limit was further improved to 10 CFU mL^−1^. [10] Using a conductive substrate-modified electrode, it has been shown that the detection limit can reach a comparable [11,12] or lower level without pre-concentration [13,14,15].

Captured with a grid column containing immuno-magnetic beads, the target Salmonella cells can be conjugated with nanocatalysts mimicking peroxidase to produce colored catalysates. The spectrophotometric detection limit of this method is approximately 10 CFU mL^−1^ [16]. Using Salmonella aptamer-conjugated upconversion nanoparticle and gold nanorods as the energy transfer donor and acceptor, respectively, this FERT fluorescence method can also accomplish a detection limit of ~10 CFU mL^−1^ [17].

The aforementioned publications report successful ultra-sensitive detection of Salmonella in food samples. Nevertheless, amplification through enzymatic reactions requires a longer time while the other methods have limited potentials in multiplex detection. Recently, biosensing techniques based on surface-enhanced Raman scattering (SERS) have attracted enormous interest due to its high sensitivity and multiplex detection capability [18,19,20,21,22,23]. A few studies integrating SERS and magnetic nanoparticles can accomplish a detection limit of 10~35 CFU mL^−1^ for Salmonella [20,21]. A dielectrophoresis (DEP) method has been employed to capture a single Salmonella cell in the sample prior to SERS detection [18]. Although the DEP method is straightforward to implement without additional assay development, the slow liquid throughput of DEP devices results in inefficient determination of pathogen cell number in milliliter volume of typical food samples, especially in multiplex detection.

Our group has successfully developed electrohydrodynamic micro-concentrators to trap a small sample aliquot (30–50 µL) containing pathogenic bacteria including Neisseria and Salmonella using centrifugal flows generated by corona discharge-induced wind [24,25]. By hanging a needle electrode with a high AC voltage (≥1 kV) of 50 kHz about 3 mm above the surface of a sample droplet on an electrically grounded slide, we can create large amounts of plasma ions at the needle tip to collide with electroneutral air molecules, generating the airflow known as ionic wind. When these air streams of ionic wind sweep across the liquid surface to generate a centrifugal vortex flow, micron-sized bacterial cells can be trapped within 20 min at the stagnant point at the center of the droplet bottom. In this study, we used an antibody-conjugated SERS tag made of Raman molecule-induced gold nanoparticle aggregates encapsulated by a silica shell, known as nanoaggregate-embedded beads (NAEBs), to recognize the trapped bacteria, Salmonella. These NAEBs have been demonstrated to provide a ultrahigh-sensitive SERS signal from even a single NAEB, due to the Raman molecule-induced “hot-spots” in the nanoaggregates. [26] Raman signals from the bound tags on bacteria can be employed to quantify pathogen cell numbers even down to the single bacterium level [18,24,25,27,28]. When a sample droplet of 30 μL was evaporated in half an hour using ionic wind to concentrate the collected bacteria, detection of a few cells of Salmonella was successfully accomplished. [25]

However, because the evaporation rate of using ionic wind is not efficient enough to dry down typical concentrated food samples of sub-milliliter volume, there remains a need to develop a rapid evaporation and concentration device to identify food pathogens such as Salmonella. In particular, the concentrated NAEBs-bound pathogen cells allow simultaneous multiplex detection by Raman spectroscopy, which is expected to have a higher throughput in multiplex detection as compared to our previous multiplex detection approach using DEP to individually count pathogen cells [18]. This report presents our preliminary results on the detection of only one pathogen. We expect the analysis time will be similar for simultaneous multiplex detection of several pathogens using the corresponding number of NAEBs tagged by different Raman reporter molecules.

Having an embedded thermoelectric heating module on a metalized glass slide, we siphoned 100 μL aliquot of SERS tag-bound Salmonella sample on the slide next to the heater. As the size of the sample droplet was rapidly reduced during the accelerated heating process, tag-bound bacterial cells were then dragged and trapped at the center of droplet bottom by ionic wind-induced centrifugal flow. Because the cells were stationed at the stagnant point on the droplet bottom when the droplet was evaporated, typical “coffee ring”-like stains of particle sediments observed in ambient and heat-assisted drying of a droplet can be avoided. When the sample is finally dried, the SERS tag-bound cells can be concentrated in one small spot to allow sensitive detection of the Raman signal. Compared with our previous electrohydrodynamic concentration device containing only a corona discharge needle, this thermoelectric evaporation-assisted device is more time-effective, with the concentrating and drying time of a 100 μL sample significantly reduced. The capability of detecting a SERS signal from a few Salmonella cells should still maintain.

We used spiked food samples including ice cubes and lettuce leaves containing a few Salmonella cells to evaluate the method feasibility of using our rapid evaporation and concentration device. Without performing cell culturing procedures to amplify Salmonella cell numbers, we filtered these samples and subsequently used ultracentrifugation steps to obtain concentrated tag-bound Salmonella samples of 200 μL. The enriched turbid sample of 100 μL was loaded onto this concentration device to dry. Finally, SERS signals from the evaporated sample in the concentration spot were detected. With the single cell detection capability [18,27,28] provided by our SERS tags, our method is expected to provide the competence to comply with the food safety regulation requirement in a timely manner without tedious bacteria cultivation procedures.

## 2. Materials and Methods

### 2.1. Materials

The same as the chip substrate in the previous paper [25], indium titanium oxide (ITO) coated conducting glass slides (7.5 cm × 2.5 cm), of which the thickness is 0.07 cm from Uni-Onward Corp. (Taipei, Taiwan, www.uni-onward.com.tw), were used to develop the miniaturized evaporation and concentration device. The following chemicals were purchased from Showa (www.showa1.com) as received for the synthesis of gold nanoparticles (AuNPs): hydrogen tetrachloroaurate trihydrate (HAuCl_4_·3H_2_O; 99.9%) and sodium citrate tribasic dihydrate (99%). Tetraethylorthosilicate (TEOS; 99.0%) and (3-aminopropyl) triethoxysilane (APTES; 99%) were obtained from Sigma-Aldrich (www.acros.com) to produce the silica-based shell of NAEB when AuNPs were encapsulated. Raman reporter molecule absorbed on AuNPs in NAEBs was Safranin O from Sigma-Aldrich. De-ionized water was made using a Milli-Q system (www.merckmillipore.com) to prepare aqueous solutions.

### 2.2. Sample Preparations

#### 2.2.1. Preparation of Antibody-Conjugated NAEBs

The procedures in previous papers were followed to prepare antibody-conjugated NAEBs [18,24,25,26,27,28]. Each NAEB, of which the size was typically from 115 to 150 nm, encapsulated 2 to 5 AuNPs to form a nanoaggregate. Raman reporter molecules such as Safranin O were adsorbed onto AuNPs to initiate a clustering process to assemble a small-sized nanoaggregate. The nanoaggregate of this size was found to possess one characteristic absorption peak at 520 nm. Safranin O-labelled NAEBs were conjugated with a monoclonal antibody mAb 4B2D, one type of IgG1 protein obtained from a mouse immunized with Salmonella Choleraesuis strain OU7085. This mAb 4B2D antibody is specific for binding with Salmonella when the phase 2 flagellin FljB is recognized, as demonstrated in our previous paper [18].

#### 2.2.2. Preparation of Spiked Salmonella Samples in De-Ionized Water

The bacteria Salmonella used in this study were heat-inactivated (56 °C, 30 min) for safety purposes. For the quantification of the bacterial concentration, the optical density of Salmonella at 600 nm in PBS solution was measured by a spectrophotometer (ChromTech CT-2300) using PBS as the reference background. Salmonella stock solutions were stored in phosphate buffer before use. Spiked water samples of various Salmonella concentrations were prepared by adding Salmonella from 2 to 20 cells into vials containing 1 mL de-ionized water. When added with an aliquot containing an excess amount of antibody-functionalized NAEBs, spiked water samples were shaken for 1 h to ensure Salmonella were bound with antibody-conjugated NAEBs. The NAEB-bound cells of Salmonella were collected by centrifuging the solution in a sample tube at 553 rcf for 15 min. The supernatant containing free unbound NAEBs was discarded using a pipette. The remaining NAEB-bound cells of Salmonella in the sample tube were re-dispersed using 200 μL de-ionized water.

#### 2.2.3. Preparation of Spiked Salmonella Samples of Ice Cubes

Ice cubes were obtained from a local convenience store. Various amounts of Salmonella from 2 to 20 cells were added to batches of 100 g ice cubes to prepare spiked samples of different Salmonella concentrations. When these ice cubes in the samples were melted, each sample solution was fed into a stainless steel filtration funnel to flow through a membrane filter of mixed cellulose esters clamped at the end of the funnel to accomplish filtration using gravity force. The filtration funnel (SS-0289) and the filter membrane (pore size 0.45 μm) were from Finetech Research and Innovation Co. (New Taipei City, Taiwan, www.finetech-filter.com). Salmonella cells remained on the membrane filter were then collected by washing with 1 mL de-ionized water. To the collected solution of 1 mL, an aliquot containing an excess amount of antibody-functionalized NAEBs was added and the resulting solution was shaken for 1 h to ensure Salmonella cells were bound with antibody-conjugated NAEBs. The aforementioned procedures in Section 2.2.2 using the ultra-centrifugation method were followed to obtain enriched samples (ca. 200 μL).

### 2.3. Preparation of Spiked Salmonella Samples of Lettuce Leaves

Lettuce leaves in a ready-to-eat salad box were obtained from a local convenience store. Various amounts of Salmonella from 4 to 20 cells were added to batches of 50 g lettuce leaves to prepare spiked samples of different Salmonella concentrations. Each lettuce leaf sample was placed in a plastic bag filled with 450 mL de-ionized water. The bags were shaken vigorously to wash Salmonella cells out from the lettuce leaves. Each washing solution was loaded into a syringe filtration device (A1-1 PureTech™) equipped with a membrane filter (pore size 5.0 μm) from Finetech Research and Innovation Co. (New Taipei City, Taiwan, www.finetech-filter.com). When the syringe piston was pressed by hands, the solution was pushed through the filter membrane to accomplish the initial filtration step to remove small leaf debris. Next, the filtrate solution containing Salmonella cells was filtered using a stainless steel filtration funnel (SS-0289) equipped with a membrane filter of mixed cellulose esters (pore size 0.45 μm), also from Finetech Research and Innovation Co., with the assistance of vacuum suction. The Salmonella cells stayed on the filter membrane were collected by washing with 10 mL de-ionized water. The collected washing solution was again filtered using the same filtration apparatus and the filter membrane was washed with just 1 mL de-ionized water to reduce sample volume. To the final collected solution of 1 mL, an aliquot containing excess amount of antibody-functionalized NAEBs was added and the resulting solution was shaken for 1 h to ensure Salmonella cells were bound with antibody-conjugated NAEBs. The aforementioned procedures in Section 2.2.2 using ultra-centrifugation method were followed to obtain enriched samples (ca. 200 μL).

### 2.4. Equipmental Set-Up

**Figure 1:**
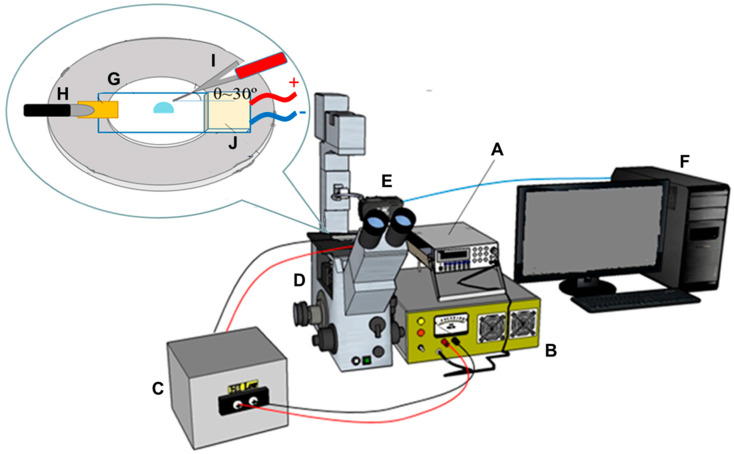
The schematic illustration of experimental set-up for the evaporation and concentration of bacteria sample using ionic wind-induced centrifugal flows enhanced with thermoelectric drying. (**A**) functional generator; (**B**) power amplifier; (**C**) transformer; (**D**) inverted-view microscope; (**E**) CCD camera; (**F**) personal computer. The top embedded graph shows the zoom-in graph of evaporation and concentration device, (**G**) containing a sessile sample droplet on a glass slide. One piece of copper ribbon electrode (**H**) is attached on one end of the slide. One corona needle (**I**) inclined at the angle 30° is hanging above the reservoir. One thermoelectric heating chip (**J**) is adhered on the other end of slide. The device is stationed on the microscope stage as pointed by the zoom-in graph.

As shown in Figure 1, except the evaporation and concentration device embedded with a thermoelectric heating unit, this study used the same set of apparatus as that in our previous paper. [25] Briefly, a function generator from Agilent Technologies (www.agilent.com) provided the ac output waveform of 50 kHz (0.5–1.0 V), pumped to ∼1000 V_rms_ via a two-stage amplification system containing one broadband voltage amplifier and a transformer, which was obtained from Industrial Testing Equipment Co. (Port Washington, NY, USA, www.industrialtest.com). The final output cord of the amplification system providing ∼1000 V_rms_ was connected to the corona needle of platinum wire (0.5 cm) to produce ionic wind. Similar to the conditions used in previous works [24,25], the optimized corona parameters were as follows. A platinum needle wire was inclined at 30-degree angle from the slide surface level and off the droplet center, pointing tangentially near the edge of the droplet. Besides, this wire was stationed 3 mm above the loaded droplet on the device slide.

The evaporation and concentration device in this study comprised one ITO coated conducting glass slide. One small piece of copper ribbon electrode was attached on one end of this slide as an electrical contact with the ground line of power source. In addition, one thermoelectric heating chip (TEC1-049.03), from Tande Energy and Temperature Associates Co. (New Taipei City, Taiwan, www.tande.com.tw), was adhered on the other end of the same conducting slide using thermal pad (TG-A6200) from T-Global Technology Co. (Taoyuan, Taiwan, www.tglobalcorp.com) to accelerate the evaporation rate of the sample droplet. The area and thickness of this heating chip were 25 × 25 mm and 4.7 mm, respectively. This device to evaporate and concentrate Salmonella sample was clamped at the stage of one inverted-view microscope (IX51, Olympus Corp., Tokyo, Japan, www.olympus-global.com). One CCD camera (ProgRes^®^, Jenoptik, Germany, www.jenoptik.com) was used to acquire microscopic images, which were then stored in a personal computer.

### 2.5. Evaporation and Concentration of Salmonella Samples

**Figure A1:**
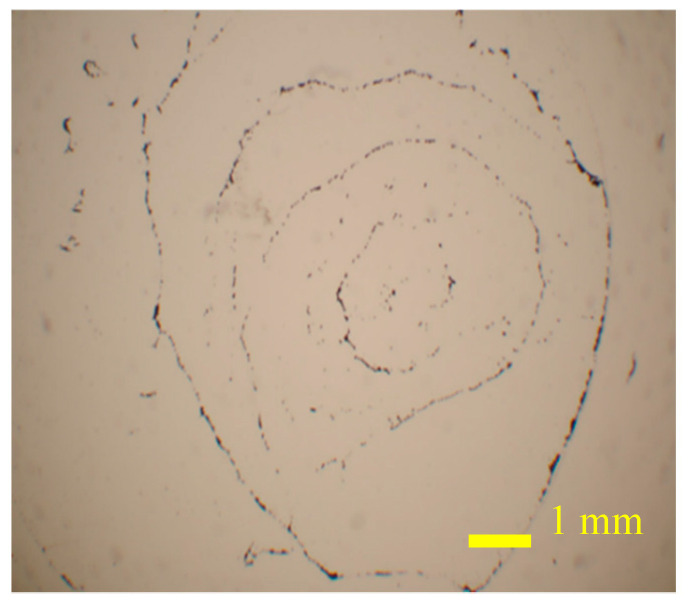
Photographic image showing dried stains of 100 μL water droplet containing 5 μm polystyrene microspheres on a glass slide, placed on a hot plate heating device to evaporate the droplet.

**Figure A2:**
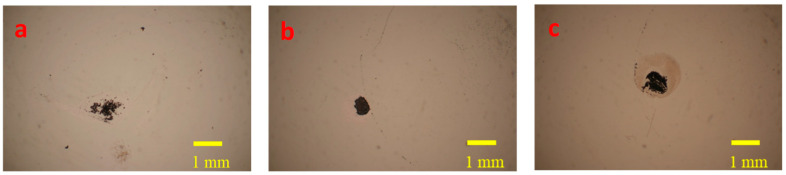
Dried stain images of 100 μL droplet containing 5 μm polystyrene microspheres, loaded on the concentration device of this study, evaporated at (**a**) 35 °C for 28 min; (**b**) 40 °C for 20 min; (**c**) 55 °C for 18 min.

Firstly, we loaded 100 μL water samples containing polystyrene microspheres (5 μm in diameter) to observe the evaporation and concentration processes when the droplet location was heated to 35 °C, 40 °C, and 55 °C. As shown in the Appendix A section, unlike the image showing coffee ring stains of microsphere sediments obtained using direct heating device (Figure A1), each image in Figure A2a–c obtained using our device at the aforementioned heating temperatures, respectively, shows one spot of concentrated microspheres. The evaporation time reduced from 28 to 20 min when the heating temperature increased from 35 to 40 °C. Although the evaporation time could be further marginally reduced at higher temperature, overheating caused charred marks as appeared in Figure A2c. Therefore, we chose 40 °C as the optimum heating temperature to concentrate Salmonella samples.

In an air conditioning laboratory at 25 °C and 50% relative humidity, when one aliquot of 100-μL enriched sample containing NAEB-bound Salmonella cells was dropped onto an ITO slide next to the thermoelectric heating unit with a pipette, it formed a hemispheric sessile droplet. As the slide was pre-heated to 40 °C, this sample droplet immediately started to be evaporated and began to shrink. At the same time, the corona needle with ac voltage produced ionic wind streams from the wire tip. Similar to our previous observations, when the evaporating droplet decreased to a size that was significantly smaller than its original size, the observed centrifugal vortex flows dragged tag-bound Salmonella cells downwards, which gradually accumulated near the center of droplet bottom [25]. With the assistance of thermoelectric heating, the evaporation rate of ionic wind-impinged droplets was accelerated. The droplet soon became a liquid film and further contracted inwards. Finally, this droplet was dried as a spot-like deposit containing the concentrated bacterial cells. The typical time span to complete the whole concentration process was 30 min. The photo-microscopic images of dried Salmonella spots were acquired. When necessary, the dried spot was colored with using Gram coloring method to enhance visualization effects. As described in Section 2.3, this evaporation and concentration device was stationed on the stage of one inverted-view microscope. One CCD camera was used to videotape the process of drying the sample droplet. When the sample was finally dried to concentrate the bacterial cells, the SERS spectra of Safranin O in NAEBs were acquired from the deposited area using a micro-Raman spectroscope (XploRA, Horiba Scientific, Kyoto, Japan, www.horiba.com). These SERS spectra were obtained using a 10X objective when the laser light beam at 638 nm stroke on the sample spot.

## 3. Results and Discussion

### 3.1. Droplet Shrinking and Drying Processes

**Figure 2:**
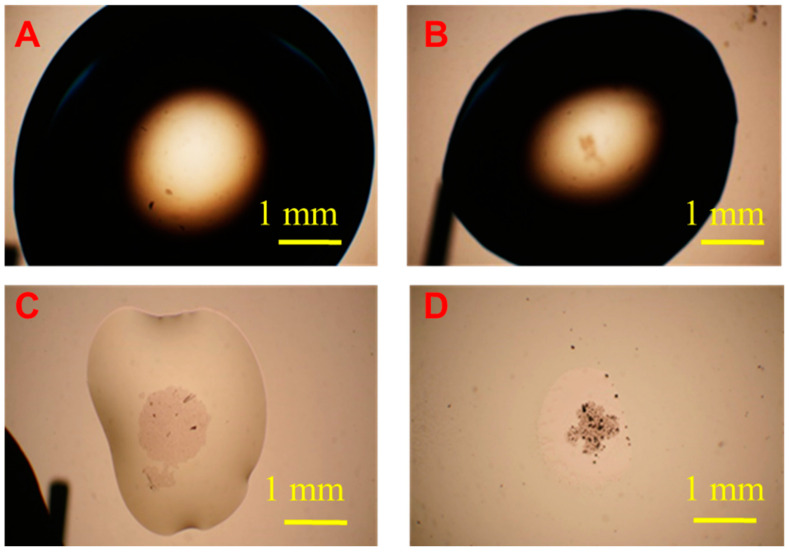
Snapshots of micro-photographic image showing the shape evolution of 100 μL sample droplet containing 1000 CFU/mL Salmonella during drying processes taken in (**A**) 0 min. (original hemispherical droplet, dia. ~9 mm); (**B**) 13 min. (evaporating hemispherical droplet, dia ~4.5 mm); (**C**) 23 min. (flatten droplet as a film, dia ~2 mm); (**D**) 30 min. (dried spot). Colony forming units, CFU.

The whole evaporation and concentration process using our thermoelectric heater-embedded micro centrifugation device takes about 30 min to obtain the dried spot containing concentrated bacterial cells. The images in Figure 2 show the shape evolution of a sample droplet during the drying processes. The loaded sessile droplet of 100 μL containing 1000 CFU/mL Salmonella shrank from dia. ~6 mm (Image A) to dia. ~4.6 mm (Image B) in 13 min because of sufficient thermoelectric heating. The side of the droplet closer to the heater evaporated more rapidly than the other side because it received heat more efficiently. Therefore, similar to our previous observations of only using ionic wind to dry down a sample droplet, the evaporation rate along the droplet surface varied, resulting in the collapse of the droplet as a film (dia. ~2 mm) in 23 min after the evaporation process. At the same time, the ionic wind-induced micro-centrifugal flow transported the NAEB-bound Salmonella cells to the center of droplet bottom (Image C) and finally, a spot-like dried deposit was obtained (Image D). When one side of droplet evaporates more rapidly than the other side, the resulted internal flow circulation can drive the NAEB microparticles to self-assemble at the surface. [29] Remaining free unbound NAEB particles accumulated at the surface alter the surface tension. The droplet is, therefore, stretched to collapse.

### 3.2. Determination of Concentrated Salmonella Cells Bound with Antibody-Conjugated NAEBs

#### 3.2.1. Determination of Salmonella in Spiked Water Samples

**Figure 3:**
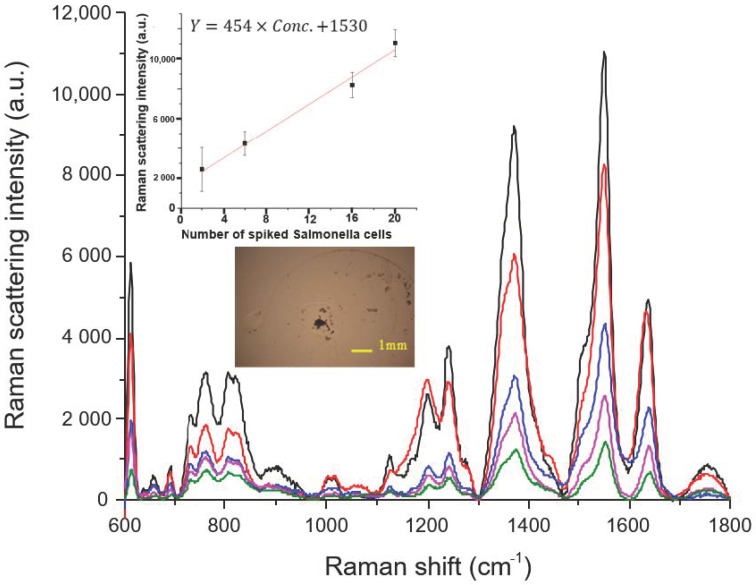
SERS spectra of Safranin O-labelled nanoaggregate-embedded beads (NAEBs) acquired from the dried spots of tag-bound Salmonella cells from spiked samples of 1 mL de-ionized water. From the top of main frame, the spectral traces in black, red, blue, and pink are from the spiked samples containing 20, 16, 6, and 2 Salmonella cells, respectively. The bottom trace in green is from the negative control sample containing only NAEB tags in the absence of Salmonella cells.

The traces in Figure 3 show the SERS spectra of Safranin O-labelled NAEBs acquired from the dried spots of tag-bound Salmonella cells. The amount of Salmonella spiked in these samples prepared using 1 mL de-ionized water are from 2 to 20 cells. Each spiked sample is added with antibody-conjugated NAEBs and enriched to ca. 200 μL from 1 mL solution using ultra-centrifugation where the supernatant liquid is removed. One aliquot of 100 μL enriched sample is pipetted on the concentration device to be dried. The embedded micro-photographic image in Figure 3 shows the dried spot of concentrated sample, spiked with six cells of Salmonella. The characteristic peaks of Safranin O marked at 615, 759, 1202, 1377, 1556, and 1641cm^−1^ noticeably appear in each trace of Figure 3 [30,31]. The assignments of these peaks to each corresponding vibration modes are listed in the Appendix B section. The pink trace of an aqueous sample containing only two cells of Salmonella can be clearly distinguished from the bottom trace in green color using a blank aliquot containing only antibody-conjugated NAEBs labelled with Safranin O in the absence of Salmonella cells. Ideally, the green trace of the blank should be close to the background Raman intensity. Although quite reproducible in intensity, the non-negligible characteristic peaks of Safranin O in the green trace of the blank suggest that some free unbound NAEBs remain in the sediment after centrifugation. The causes for this are still under investigation and one possibility is that some NAEBs aggregated, resulting in heavier particles not being removed by centrifugation. Our preliminary results using dextran-coated NAEBs show smaller blank signals, indicating that an anti-nonspecific adsorption coating on NAEBs minimizes aggregation of NAEBs. The spectral traces in Figure 3 also show that the peak intensity increased when the spiked amount of Salmonella cells increases. These findings are consistent with the results of our previous study of detecting Salmonella using SERS tags with an ionic wind-based micro-centrifugal concentration device [25].

Each sample was firstly enriched to ca. 200 μL and one aliquot of 100 μL was siphoned to pipette onto the concentration device. Therefore, in Figure 3, the pink traces are from just two NAEB-bound Salmonella cells. This finding is consistent with the results in our previous study of using NAEB Raman tags to detect a single cell of Salmonella [25]. Therefore, there should be no considerable sample loss during the aforementioned enrichment procedures.

Using the height intensity of the 1556 cm^−1^ peak of each sample, one standard curve was established, as shown in the inset of Figure 3. This curve indicates a highly linear relationship (R = 0.99) between the Raman intensity and number of Salmonella cells in the spiked samples, where two replicates were determined for each sample.

#### 3.2.2. Determination of Salmonella in Spiked Ice Cube Samples

**Figure 4:**
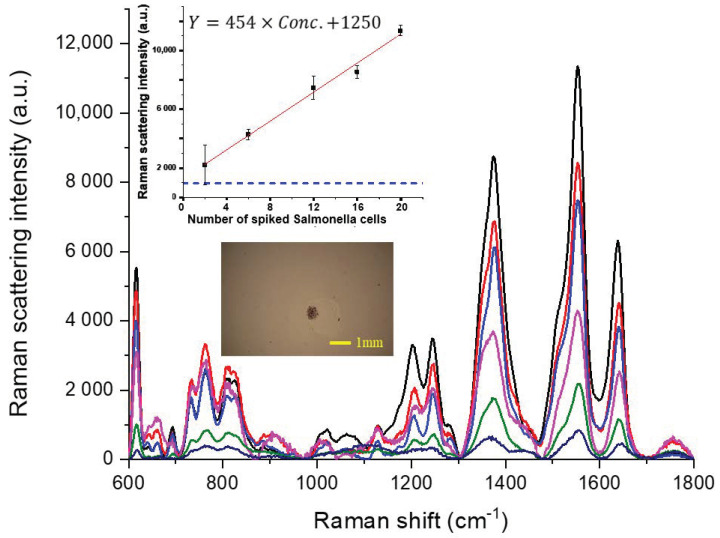
SERS spectra of Safranin O-labelled NAEBs acquired from the dried spots of tag-bound Salmonella cells from spiked samples of 100 g ice cubes. From the top of main frame, the spectral traces in black, red, blue, pink, and green are from the spiked samples containing 20, 16, 12, 6, and 2 Salmonella cells, respectively. The bottom trace in green is from the negative control sample containing only NAEB tags in the absence of Salmonella cells.

Figure 4 contains the SERS traces of Safranin O-labelled NAEB tags acquired from the tag-bound Salmonella cells in the dried spots of ice cube samples of 100 g, in which various amounts of Salmonella from 2 to 20 cells were spiked. After a spiked ice cube sample was melted and filtered, the Salmonella cells remaining on the filter were collected by washing with 1 mL de-ionized water. Then, each sample of the collected solution was added with antibody-conjugated NAEBs and enriched to ca. 200 μL from 1 mL using ultra-centrifugation where the supernatant liquid was removed. One aliquot of 100 μL enriched sample was pipetted onto the concentration device to be dried. The embedded photographic image in Figure 4 shows the dried spot of the concentrated ice cube sample, spiked with six cells of Salmonella. Similar to the spectra in Figure 3, the green trace of the spiked sample containing only two cells of Salmonella can also be distinguished from the bottom trace in indigo, using an aliquot containing only antibody-conjugated NAEBs labelled with Safranin O in the absence of Salmonella cells. In addition to clear characteristic peaks of Safranin O in these spectra in Figure 4, the height intensities of each trace at 1556 cm^−1^ are comparable with that of 1 mL de-ionized water samples spiked with the same amount of Salmonella cells in Figure 3. Using the peak intensities at 1556 cm^−1^ corresponding to 2, 6, 12, 16, and 20 cells in Figure 4, we also obtained a linear relationship (R = 0.985) between Raman intensity and number of Salmonella cells in the spiked samples, as shown in the inset of Figure 4, where two replicates were determined for each sample. The Raman intensity of the blanks was reproducible with a value of 860 ± 35. To avoid a false-positive signal due to the background signal, we define the background level (shown as a dashed line in Figure 3) as the mean plus 3-times the standard deviation of the Raman intensity from the blanks. It can be seen that the signal from the sample with only two Salmonella cells is well above the background level.

If sample loss possibly occurs in the filtration and collection procedures for the ice cube samples spiked with only two Salmonella cells, the signal intensity should vary significantly with some samples exhibiting a SERS intensity below the background level because some sample aliquots may contain no Salmonella cells. The reasonable reproducibility at the point of two cells in the aforementioned standard curve suggests that the sample loss is negligible while the method achieves the capability of detecting a SERS signal from a single cell. The safety requirement of drinking water is that no Salmonella should be found in the 100 mL cultured sample. When an ice cube sample in street drinks or beverages was determined, the detection limit of our method nearly reached the regulation compliance standard without cultivation procedures.

#### 3.2.3. Determination of Salmonella in Spiked Lettuce Leaf Samples

**Figure 5:**
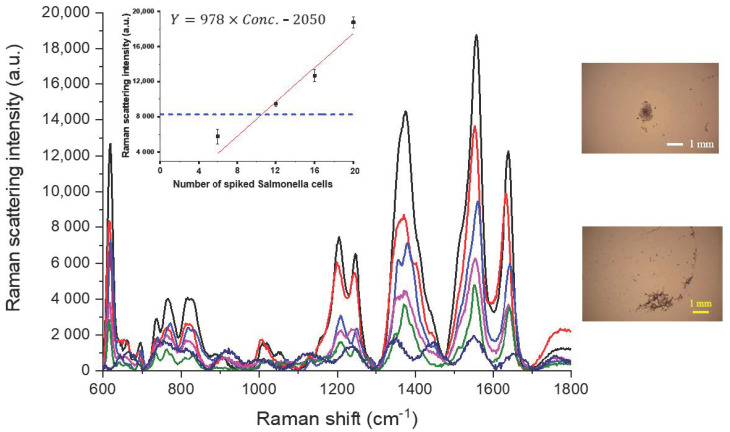
SERS spectra of Safranin O-labelled NAEBs acquired from the dried spots of tag-bound Salmonella cells from spiked samples of 50 g of lettuce leaves. From the top of the main frame, the spectral traces in black, red, blue, and pink are from the spiked samples containing 20, 16, 12, and 6 Salmonella cells, respectively. The second to bottom trace in green and the bottom trace in indigo, respectively, are from a negative control sample in the absence of Salmonella cells and a blank in the absence of Salmonella cells and NAEBs.

Figure 5 shows the clear SERS traces of Safranin O-labelled NAEBs from the tag-bound Salmonella cells in the dried spots of spiked lettuce leaf samples. The spiked amounts in these samples of 50 g lettuce leaves were 6, 12, 16, and 20 cells. In Figure 5, the second to bottom and bottom traces in green and indigo, respectively, used a negative control sample containing Safranin O-labelled NAEBs but no Salmonella cells and a blank containing only lettuce leaves but without Safranin O-labelled NAEBs and Salmonella cells. When a sample solution of 450 mL de-ionized water containing spiked lettuce leaves was filtered, the bacterial cells that remained on the filter membrane were collected by washing with 1 mL de-ionized water. Then, each sample of collected solution was enriched to ca. 200 μL from 1 mL using ultra-centrifugation where the supernatant liquid was removed. One aliquot of 100 μL enriched sample was pipetted onto the concentration device to be dried. The upper embedded micro-photographic image in Figure 5 next to the main frame shows the dried concentrated sample of lettuce leaf washing solution, spiked with 6 Salmonella cells. Using the peak intensities at 1556 cm^−1^ corresponding to 6, 12, 16, and 20 cells in Figure 5, one standard calibration curve was obtained, as shown in the inset of Figure 5. The intensity values of these samples are somewhat higher than that with the same number of cells in Figure 3 or Figure 4. However, the linearity of the standard curve between Raman intensity and number of Salmonella cells in the spiked samples deteriorated to a fairly acceptable level (R = 0.97), when two replicates were determined for each sample.

Using a lettuce leaf sample as a blank containing no Salmonella cells and Safranin O-labelled NAEBs, the Raman spectrum as shown in the bottom indigo trace of Figure 5 does not contain spectral peak features of Safranin O. In addition, the intensities around the characteristic peaks of Safranin O are much lower than that of the negative control sample, as shown in the second bottom green trace. For the negative control sample, as shown in the lower micro-photographic image of Figure 5 next to the main frame, some fibrous tiny residues of lettuce leaves were also found to sediment on the slide, although not in the same location of trapped bacterial cells. The peak intensity at 1556 cm^−1^ in the second to bottom green trace in Figure 5 was about 5000 counts, higher than the intensities (≤2000 counts) of other negative control samples without the fibrous residues, as shown in Figure 3 and Figure 4, indicating the possibility of nonspecific adsorption of NAEBs onto the fibrous residues. Hence, it is important to minimize the anti-nonspecific adsorption property of the NAEBs by an antifouling coating in order to lower the detection limit. Because the Raman intensity of the negative control samples (2985 ± 1766) varies highly, we defined the background level (shown as a dashed line in Figure 5) as the mean plus 3-times the standard deviation of the Raman intensity from the negative control samples and accepted only the results above the background level as positive results. By this definition, we estimated that the detection limit of determining spiked lettuce leaf sample was 12 Salmonella cells per 50 g.

The safety requirement of ready-to-eat lettuce is that no Salmonella should be found in the cultured sample of 20 g leaves. Because of the problem of residue absorption of NAEBs onto lettuce leaves, our detection limit of 5 Salmonella cells per 20 g lettuce leaves is slightly higher than the regulation compliance standard. When our method was employed to determine real samples, in addition to increasing the sampling weight of lettuce leaves to 50 g, it was important to further improve the anti-nonspecific adsorption property of the NAEB surface.

## 4. Conclusions

We have successfully developed a miniaturized rapid evaporation and concentration device via integrating a thermoelectric heating unit with ionic wind-induced droplet centrifugation chip to determine spiked Salmonella in food samples without bacteria cultivation steps. The loaded sample aliquot of ~100 μL containing Raman tag-bound Salmonella cells can be dried in 30 min because of the efficient evaporation provided by thermoelectric heater. At the same time, Salmonella cells were dragged toward to the center of sample droplet bottom by ionic wind-induced centrifugal flows to concentrate in a dried spot. Single cell detection capability is provided by the sensitive scattering signals acquired from concentrated Raman tag-bound Salmonella deposits. Using SERS detection, we demonstrated that this device can detect 2 and 12 Salmonella cells spiked in a 100 g ice cube sample and 50 g lettuce leaf sample, respectively. With this high sensitivity, the detection limit of our method is close to the food safety regulation compliance standard, while tedious bacteria cultivation is not required.

Using this simple device, rapid sample concentration can be accomplished by a nontechnical person simply by loading a sample droplet using a pipette. The evaporation and concentration efficiency of this device should be further improved through combining a Marangoni effect-based technique such as a superhydrophobic surface coating with surfactant or viscosity modifier addition. The contact line pinning of the sample droplet will be controlled more precisely during the evaporation process to avoid overheating at high temperature. Tag-bound Salmonella cells could precipitate in a more concentrated manner at the bottom of a conical well when the droplet drying is completed. We will investigate the aforementioned ideas in the future.

## Appendix B

**Table A1:** Assignment of Vibrational Bands in SERS Spectra of Safranin O.

SERS (cm^−1^)	Assignment
615	δ(C–C–C)
759	ω(NH_2_) + γ(C–C–C)
1202	υ(N–C) + δ(H-C-C)
1377	υ(C–C)
1556	υ(C–C)
1641	δ(NH_2_) + υ(C–C)

δ, in plane bending; γ, out of plane bending; ω, wagging; υ, stretching.

## References

[1] (2018). Compendium of Microbiological Criteria for Food.

[2] Du X.-J., Zhou T.-J., Li P., Wang S. (2017). A rapid Salmonella detection method involving thermophilic helicase-dependent amplification and a lateral flow assay. Mol. Cell. Probes.

[3] Dao T.N.T., Yoon J., Jin C.E., Koo B., Han K., Shin Y., Lee T.Y. (2018). Rapid and sensitive detection of Salmonella based on microfluidic enrichment with a label-free nanobiosensing platform. Sens. Actuators B Chem..

[4] Hu J., Huang R., Sun Y., Wei X., Wang Y., Jiang C., Geng Y., Sun X., Jing J., Gao H. (2019). Sensitive and rapid visual detection of *Salmonella Typhimurium* in milk based on recombinase polymerase amplification with lateral flow dipsticks. J. Microbiol. Methods.

[5] Zhang Y., Tian J., Li K., Tian H., Xu W. (2019). Label-free visual biosensor based on cascade amplification for the detection of Salmonella. Anal. Chim. Acta.

[6] Rosen D.K., Gallardo M., Vail M., Hellberg R.S. (2020). Microplate immunocapture coupled with the 3M molecular detection system and selective plating for the rapid detection of *Salmonella infantis* in dry dog food and treats. J. Microbiol. Methods.

[7] Cinti S., Volpe G., Piermarini S., Delibato E., Palleschi G. (2017). Electrochemical Biosensors for Rapid Detection of Foodborne Salmonella: A Critical Overview. Sensors.

[8] Silva N.F.D., Magalhães J.M.C.S., Freire C., Delerue-Matos C. (2018). Electrochemical biosensors for Salmonella: State of the art and challenges in food safety assessment. Biosens. Bioelectron..

[9] Zhu W., Chen Y., He Y., Fang W., Ying Y., Li Y., Fu Y. (2020). Cooperation Mode of Outer Surface and Inner Space of Nanochannel: Separation-Detection System Based on Integrated Nanochannel Electrode for Rapid and Facile Detection of Salmonella. Anal. Chem..

[10] Hou Y., Tang W., Qi W., Guo X., Lin J. (2020). An ultrasensitive biosensor for fast detection of Salmonella using 3D magnetic grid separation and urease catalysis. Biosens. Bioelectron..

[11] Dinshaw I.J., Muniandy S., Teh S.J., Ibrahim F., Leo B.F., Thong K.L. (2017). Development of an aptasensor using reduced graphene oxide chitosan complex to detect Salmonella. J. Electroanal. Chem..

[12] Appaturi J.N., Pulingam T., Thong K.L., Muniandy S., Ahmad N., Leo B.F. (2020). Rapid and sensitive detection of Salmonella with reduced graphene oxide-carbon nanotube based electrochemical aptasensor. Anal. Biochem..

[13] Lu D., Pang G., Xie J. (2017). A new phosphothreonine lyase electrochemical immunosensor for detecting Salmonella based on horseradish peroxidase/GNPs-thionine/chitosan. Biomed. Microdevices.

[14] Dai G., Li Z., Luo F., Ai S., Chen B., Wang Q. (2019). Electrochemical determination of *Salmonella typhimurium* by using aptamer-loaded gold nanoparticles and a composite prepared from a metal-organic framework (type UiO-67) and graphene. Microchim. Acta.

[15] Ye Y., Yan W., Liu Y., He S., Cao X., Xu X., Zheng H., Gunasekaran S. (2019). Electrochemical detection of Salmonella using an invA genosensor on polypyrrole-reduced graphene oxide modified glassy carbon electrode and AuNPs-horseradish peroxidase-streptavidin as nanotag. Anal. Chim. Acta.

[16] Wang L., Huo X., Zheng L., Cai G., Wang Y., Liu N., Wang M., Lin J. (2020). An ultrasensitive biosensor for colorimetric detection of Salmonella in large-volume sample using magnetic grid separation and platinum loaded zeolitic imidazolate Framework-8 nanocatalysts. Biosens. Bioelectron..

[17] Cheng K., Zhang J., Zhang L., Wang L., Chen H. (2017). Aptamer biosensor for *Salmonella typhimurium* detection based on luminescence energy transfer from Mn^2+^-doped NaYF4:Yb, Tm upconverting nanoparticles to gold nanorods. Spectrochim. Acta Part A Mol. Biomol. Spectrosc..

[18] Lin H.-Y., Huang C.-H., Hsieh W.-H., Liu L.-H., Lin Y.-C., Chu C.-C., Wang S.-T., Kuo I.T., Chau L.-K., Yang C.-Y. (2014). On-line SERS Detection of Single Bacterium Using Novel SERS Nanoprobes and A Microfluidic Dielectrophoresis Device. Small.

[19] Faulds K., Jarvis R., Smith W.E., Graham D., Goodacre R. (2008). Multiplexed detection of six labelled oligonucleotides using surface enhanced resonance Raman scattering (SERRS). Analyst.

[20] Zhang H., Ma X., Liu Y., Duan N., Wu S., Wang Z., Xu B. (2015). Gold nanoparticles enhanced SERS aptasensor for the simultaneous detection of *Salmonella typhimurium* and Staphylococcus aureus. Biosens. Bioelectron..

[21] Kearns H., Goodacre R., Jamieson L.E., Graham D., Faulds K. (2017). SERS Detection of Multiple Antimicrobial-Resistant Pathogens Using Nanosensors. Anal. Chem..

[22] Sánchez-Purrà M., Carré-Camps M., de Puig H., Bosch I., Gehrke L., Hamad-Schifferli K. (2017). Surface-Enhanced Raman Spectroscopy-Based Sandwich Immunoassays for Multiplexed Detection of Zika and Dengue Viral Biomarkers. ACS Infect. Dis..

[23] Cheng Z., Choi N., Wang R., Lee S., Moon K.C., Yoon S.-Y., Chen L., Choo J. (2017). Simultaneous Detection of Dual Prostate Specific Antigens Using Surface-Enhanced Raman Scattering-Based Immunoassay for Accurate Diagnosis of Prostate Cancer. ACS Nano.

[24] Chen Y.-Y., Fang Y.-C., Lin S.-Y., Lin Y.-J., Yen S.-Y., Huang C.-H., Yang C.-Y., Chau L.-K., Wang S.-C. (2017). Corona-induced micro-centrifugal flows for concentration of Neisseria and Salmonella bacteria prior to their quantitation using antibody-functionalized SERS-reporter nanobeads. Microchim. Acta.

[25] Su S.-R., Chen Y.-Y., Li K.-Y., Fang Y.-C., Wang C.-H., Yang C.-Y., Chau L.-K., Wang S.-C. (2019). Electrohydrodynamically enhanced drying droplets for concentration of Salmonella bacteria prior to their detections using antibody-functionalized SERS-reporter submicron beads. Sens. Actuators B Chem..

[26] Huang P.-J., Chau L.-K., Yang T.-S., Tay L.-L., Lin T.-T. (2009). Nanoaggregate-Embedded Beads as Novel Raman Labels for Biodetection. Adv. Funct. Mater..

[27] Huang P.-J., Tay L.-L., Tanha J., Ryan S., Chau L.-K. (2009). Single-Domain Antibody-Conjugated Nanoaggregate-Embedded Beads for Targeted Detection of Pathogenic Bacteria. Chem. Eur. J..

[28] Tay L.-L., Huang P.-J., Tanha J., Ryan S., Wu X., Hulse J., Chau L.-K. (2012). Silica encapsulated SERS nanoprobe conjugated to the bacteriophage tailspike protein for targeted detection of Salmonella. Chem. Commun..

[29] Pathak B., Hatte S., Basu S. (2017). Evaporation Dynamics of Mixed-Nanocolloidal Sessile Droplets. Langmuir.

[30] Lofrumento C., Arci F., Carlesi S., Ricci M., Castellucci E., Becucci M. (2015). Safranin-O dye in the ground state. A study by density functional theory, Raman, SERS and infrared spectroscopy. Spectrochim. Acta Part A Mol. Biomol. Spectrosc..

[31] Sylvestre S., Sebastian S., Oudayakumar K., Jayavarthanan T., Sundaraganesan N. (2012). Experimental (FT-IR, FT-Raman and UV–Vis) spectra and theoretical DFT investigations of 2,3-diaminophenazine. Spectrochim. Acta Part A Mol. Biomol. Spectrosc..

